# From twitch to relaxation: Obesity dysregulates muscle contractile function

**DOI:** 10.14814/phy2.70731

**Published:** 2026-02-15

**Authors:** L. Cesanelli, H. Degens, P. Minderis, D. Satkunskiene

**Affiliations:** ^1^ Institute of Sport Science and Innovations, Lithuanian Sports University Kaunas Lithuania; ^2^ Department of Life Sciences Manchester Metropolitan University Manchester UK; ^3^ Department of Health Promotion and Rehabilitation Lithuanian Sports University Kaunas Lithuania

**Keywords:** contractility, mechanobiology, muscle physiology, musculoskeletal disease, obesity

## Abstract

Obesity has been increasingly recognized not only as a metabolic disorder but also as a condition that impairs neuromuscular function, including strength relative to body mass. This translational study investigated whether obesity affects both force generation and contraction‐relaxation dynamics. In control (CN) and diet‐induced obese (OB) male mice, contractile properties of isolated extensor digitorum longus (EDL) and soleus (SOL) muscles were assessed in vitro. In parallel, plantar flexor performance was assessed in 25 normal‐weight (CN) and 25 class I obese (OB) sedentary men through maximal voluntary isometric contractions and a dynamic calf raise test. OB mice exhibited lower specific force and slower rates of force development and relaxation in both EDL and SOL (*p* < 0.05). In men, the lower rate of torque development and prolonged relaxation kinetics of the plantar flexors (*p* < 0.05), combined with a higher body mass to maximal voluntary isometric torque ratio (*p* < 0.05), contributed to slower calf raise phases in OB compared to CN men (*p* < 0.05). These findings reveal that obesity not only has a negative impact on the muscle force generating capacity but also induces slower muscle contractile kinetics.

## 1 INTRODUCTION

Obesity is widely recognized as a significant public health concern, associated not only with metabolic and cardiovascular disorders but also with profound alterations in neuromuscular function (Balasundaram & Daley, 2025; Mehta, 2015; O'Brien et al., 2017; Tallis et al., 2018; Tomlinson et al., 2016). These impairments are not merely due to the passive effect of excess body mass but also involve intrinsic dysfunctions in muscle contractility, fatigue resistance, and neuromuscular coordination, with negative consequences for balance, gait, and overall mobility (Cesanelli, Degens, et al., 2025; Mehta, 2015; Wearing et al., 2006).

A common observation in individuals with obesity is a lower muscle strength‐to‐body mass ratio, indicating relative functional weakness despite preserved or even increased absolute strength in antigravity muscles (Cesanelli, Degens, et al., 2025; Maffiuletti et al., 2007; Tallis et al., 2018; Tomlinson et al., 2016). This suggests that the chronic mechanical loading from excess body mass does not protect lower‐limb musculature. Potential contributors to reduced force‐generating capacity are intrinsic muscle alterations, such as increased intramuscular lipid infiltration and impaired excitation–contraction coupling (Cesanelli, Minderis, et al., 2025; O'Brien et al., 2017; Rahemi et al., 2015; Tallis et al., 2018; Wearing et al., 2006). Additionally, excess adiposity may alter tendon and extracellular matrix properties, increasing tissue viscosity, which can reduce the efficiency of force transmission and exacerbate fatigue during repetitive or dynamic tasks (Biltz et al., 2020; Cesanelli, Degens, et al., 2025; Hausman et al., 2014; Rahemi et al., 2015; Tallis et al., 2018; Tomlinson et al., 2021).

Despite these observations, most studies to date have primarily focused on the ability of muscles to generate torque or tension during voluntary or electrically induced contractions. However, force production is only one aspect of a muscle contraction cycle. The relaxation phase, during which muscle tension returns to baseline after contraction, is equally important for coordinated and efficient movement as the ability to generate force (Corcos et al., 1996; Hunter et al., 2008; Molenaar et al., 2013; Robichaud et al., 2005). Indeed, the rate of force relaxation is essential during the performance of cyclic and ballistic tasks such as walking, running, or stair climbing, and is crucial for minimizing the risk of injury or falls (Mathern et al., 2019).

From a biomechanical perspective, a slower muscle relaxation could translate into inefficient joint movement control, prolonged ground contact times, increased energy expenditure during locomotion, and decreased performance and efficiency in activities requiring high movement cadence or well‐coordinated motion (Cesanelli, Degens, et al., 2025; Mathern et al., 2019; Wearing et al., 2006). Yet, muscle relaxation remains an underexplored domain in obesity research. It is therefore important to determine whether obesity‐related muscle impairments extend beyond a relatively lower peak force generation to body mass ratio to altered force‐time dynamics during both contraction and relaxation. Because relaxation kinetics reflect both tissue energetics (e.g., intracellular calcium handling) and structuro‐mechanical properties (e.g., tissue composition and viscoelasticity)—which are disrupted in obesity (Cesanelli, Degens, et al., 2025; Cesanelli, Minderis, et al., 2025; Tallis et al., 2018)—we expected obesity to also slow the rate of relaxation. This issue is of critical importance not only from a physiological standpoint but also for its clinical and functional implications.

To address this issue, we systematically investigated how obesity affects the muscle contraction and relaxation properties in mice and humans. Using a well‐established mouse model of diet‐induced obesity, we assessed the contractile function in fast‐twitch (extensor digitorum longus, EDL) and slow‐twitch (soleus, SOL) muscles under controlled in vitro conditions. This approach enabled precise analysis of muscle force development and relaxation kinetics independent of neural input or mechanical confounders. In the parallel human study, we noninvasively evaluated plantar flexor muscle function in sedentary adult men with normal weight and class I obesity. We hypothesized that obesity would not only reduce the capacity to generate force but also slow muscle contraction and relaxation.

## 2 METHODS

### 2.1 Study design

**FIGURE 1:**
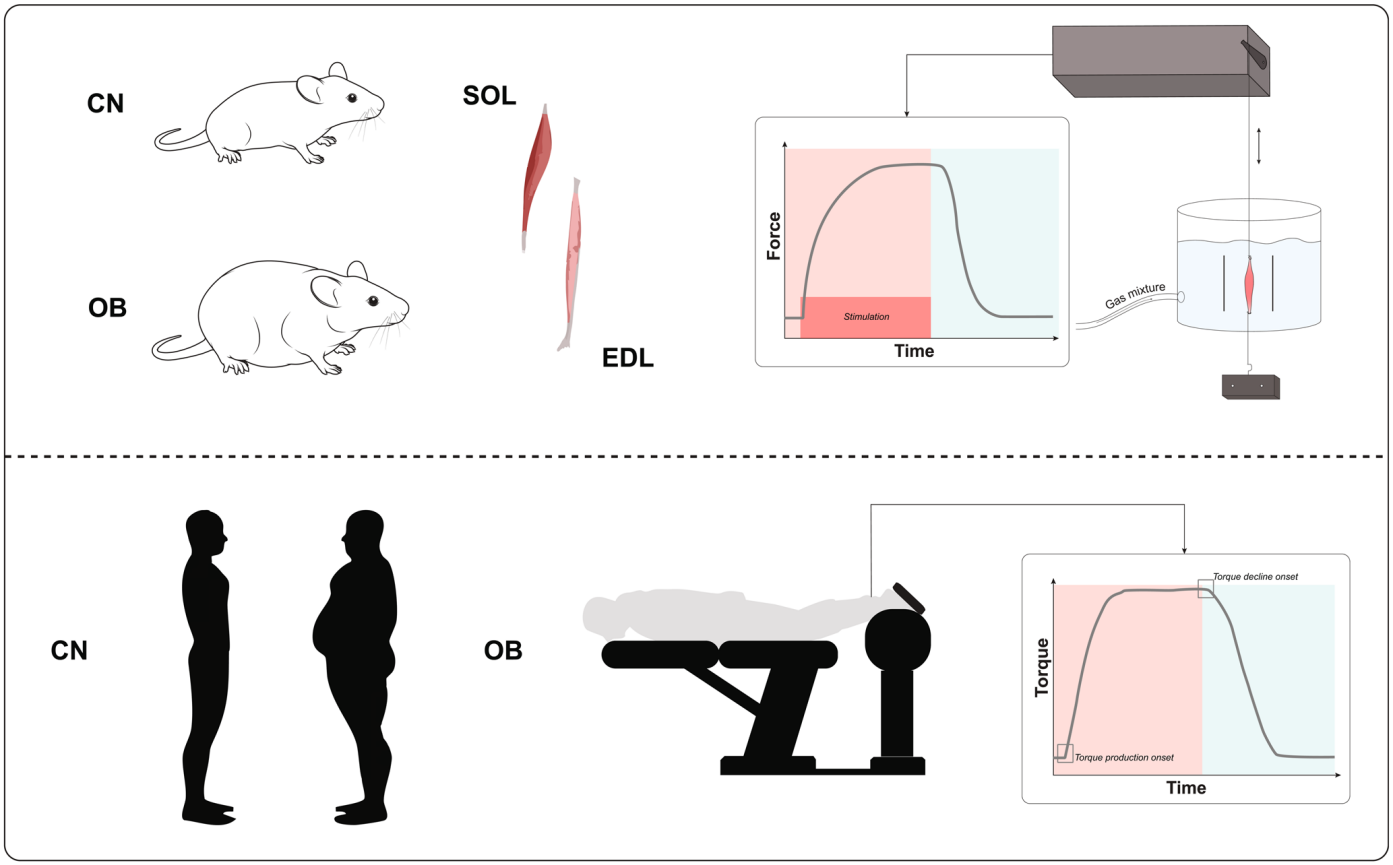
Overview of muscle contractile property assessments. Upper panel: Animal model arm showing isolated soleus (SOL) and extensor digitorum longus (EDL) muscles from control (CN) and diet‐induced obese (OB) mice subjected to electrically evoked twitches and tetani. Lower panel: Human model arm illustrating maximal voluntary plantar flexor contractions performed by normal‐weight (CN) and obese (OB) participants.

This translational study combined a mouse model of diet‐induced obesity with a cross‐sectional investigation in humans (Figure 1). A subset of data from both the animal and human studies has been previously published (Cesanelli, Degens, et al., 2025; Cesanelli, Minderis, et al., 2025).

At 10 weeks of age, male C57BL/6 mice were randomly assigned to either a high‐fat diet (60% fat, 20% carbohydrate, 20% protein by energy; Research Diets, D12492) (OB, *n* = 10) or a standard chow diet (energy, 65% carbohydrate, 24% protein, 11% fat; Altromin, 1320) (CN, *n* = 10) for 28 weeks. Mice were housed under controlled environmental conditions (12:12 h light–dark cycle, 21°C–23°C) with ad libitum access to food and water throughout the intervention period. Mice were euthanized by CO_2_ exposure following a 5‐h fast, and all procedures were conducted at room temperature (21°C–23°C). After euthanasia, the soleus (SOL) and extensor digitorum longus (EDL) muscles were excised for contractile function testing. Muscles were harvested from the right limb in all animals. Ethical approval was obtained from the Lithuanian State Food and Veterinary Service (Ref. G2‐255).

In the human study, fifty 30‐ to 50‐year‐old sedentary men volunteered: 25 with normal BMI (CN, 18.5–24.9 kg/m^2^) and 25 classified as class I obese (OB, 30.0–34.9 kg/m^2^). All participants reported no regular physical activity or structured exercise in the past 2 years. Written informed consent was obtained after providing detailed information on study aims, procedures, and potential risks. The study complied with the Declaration of Helsinki and was approved by the Kaunas Regional Biomedical Research Ethics Committee (No. 2023‐BE10‐0001). Exclusion criteria included self‐reported knee or ankle injuries, anterior knee or ankle pain, Achilles tendon rupture or tendinopathy, cardiovascular, respiratory, or neuromuscular conditions, or a history of drug abuse.

### 2.2 In vitro muscle contractile properties, in mice

Contractility of SOL and EDL muscles (*n* = 10 in each group) was examined as previously described (Cesanelli et al., 2024; Cesanelli, Minderis, et al., 2025; Fokin et al., 2019). After sacrifice, SOL and EDL muscles were excised and tied with 5–0 silk suture to the proximal and distal tendons. They were then placed in a 100‐mLRadnoti tissue bath filled with Tyrode solution (121 mM NaCl, 5 mM KCl, 0.5 mM MgCl_2_, 1.8 mM CaCl_2_, 0.4 mM NaH_2_PO_4_, 0.1 mM NaEDTA, 24 mM NaHCO_3_, 5.5 mM glucose) that was bubbled with a gas mixture of 95% O_2_ and 5% CO_2_ at pH 7.4. The bath was maintained at room temperature (~21°C–23°C) during all experiments. The muscle was suspended vertically between two platinum plate electrodes parallel to the length of the muscle in the bath, with the proximal tendon attached securely to the lever arm of the muscle test system (1200A‐LR Muscle Test System, Aurora Scientific Inc., Canada) and the distal tendon to a fixed iron hook. After mounting, a brief test stimulation was applied to verify secure attachment and confirm the absence of slippage before initiating data collection. Optimal length of the muscle (L_0_) was assessed by repeated twitch contractions until active twitch force did not increase with a further increase in muscle length. The muscle was then photographed with the length scale in the background to assess muscle length with a precision of 0.1 mm. The muscle was kept at this optimal length during the assessment of contractile properties. The muscles were subjected to two additional twitch contractions at L_0_ followed by 300‐ms (EDL) and 900‐ms (SOL) trains of stimuli at 20, 50, 80, 100, 150, and 200 Hz for assessment of peak tetanic force from a force‐frequency curve, with 30 s rest between each contraction to prevent development of muscle fatigue.

Peak twitch and tetanic (SOL: 80 Hz; EDL: 100 Hz) force‐time curves were used to analyze and extract peak force (PF), specific force (SF; force per unit physiological cross‐sectional area), early and late rate of force development as peak slope in N/s (RFD_E_, RFD_L_), respectively 0%–30% and 0%–60% of the force‐time curve between onset of contraction and peak force, and RFD/PF ratios. The force–time relaxation curves were fitted with a double exponential decay function of the form y = y_0_ + A_1_e^−k1x^ + A_2_e^−k2x^ (Cesanelli, Minderis, et al., 2025) to determine the two rate constants (k1 and k2). For twitch contractions, fitting was initiated from peak force. Tetanic contractions exhibit a “shoulder phase”, visually identified as the inflection point associated with the abrupt increase in force decay rate. Therefore, for tetanic contractions, double‐exponential fitting was applied to the relaxation phase occurring after the relaxation shoulder. The slow relaxation rate during the shoulder phase (k_slow_) of a tetanic contraction was quantified as the slope of a linear fit applied to the initial relaxation phase, from peak force to the onset of the rapid force decline. The slope was subsequently normalized to peak force (PF) according to k_slow_ = −k/PF, where k represents the slope of the linear fit (Dvornikov & Harris, 2025). Half‐relaxation time (HRT) was calculated as the time required for twitch and tetanic force to decline from peak to 50% of peak value. The muscle physiological cross‐sectional area (PCSA) was calculated by dividing muscle wet mass by the product of fiber length (Lf) and the skeletal muscle density (1.06 g /cm^3^) assuming Lf to L_0_ ratios of 0.45 and 0.70 for EDL and SOL, respectively, following established approaches for mouse skeletal muscle (Brooks & Faulkner, 1988; Cesanelli et al., 2024; Fokin et al., 2019; Minderis et al., 2016). This method provides a physiologically meaningful estimate of the force‐generating area and is widely used in animal muscle mechanics.

### 2.3 Maximal voluntary isometric contractions, in humans

Following anamnesis and familiarization, testing commenced with collecting anthropometric data, including body mass (BM) and body fat analysis using a Tanita‐305 body‐fat analyzer (Tanita Corp) and height (KaWe PERSON‐CHECK stadiometer, Kirchner & Wilhelm GMBH þ Co. KG). Once familiarized, participants were then instructed by a supervisor to contract their dominant leg plantar flexors as fast and forcefully as possible, receiving verbal encouragement during the test (Maffiuletti et al., 2016). The subjects executed three maximal contractions at a 90° ankle joint angle. All measurements were conducted using a calibrated Biodex System 4 dynamometer (Biodex Medical Systems). Each contraction lasted 2 s, with 2 min recovery between each contraction. The maximal voluntary torque (MVT) represented the peak isometric torque (Nm) during the contraction. The rate of torque development (RTD) was calculated as peak slope (Nm/s) in early (RTD_E_) (0–100 ms) and late (RTD_L_) (0–300 ms) phases of torque development (Maffiuletti et al., 2016). The onset of a contraction was defined as the moment that the torque of the plantar flexors surpassed 2.5% of the baseline‐to‐peak torque difference, ensuring a reproducible criterion while minimizing baseline noise (Maffiuletti et al., 2016). The sampling frequency was 1000 Hz. The collected torque signals were filtered using a fourth‐order low‐pass Butterworth filter (cut‐off frequency: 10 Hz) to minimize high‐frequency noise while preserving the mechanical signal characteristics, with baseline noise consistently below 1% of MVT. The RTD × MVT^−1^ and the BM × MVT^−1^ were calculated (Degens et al., 2021). The MVT and RTD from the best (highest peak torque) of the three repetitions of the voluntary isometric contractions were analyzed. The force–time relaxation curves were fitted with a double exponential decay function of the form y = y_0_ + A_1_e^−k1x^ + A_2_e^−k2x^ (Cesanelli, Minderis, et al., 2025) to determine the two rate constants (k1 and k2); fitting was initiated from peak torque. K_slow_ was not calculated for maximal voluntary contractions due to the absence of a distinct “shoulder phase” seen in mouse titanic contractions. Half‐relaxation time (HRT) was calculated as the time required for torque to decline from peak to 50% of peak value.

### 2.4 Calf raise test

For the calf raise test (CRT), participants were instructed to perform as many calf raises as possible at a self‐determined pace in 30 s (Hébert‐Losier et al., 2009). These test conditions were chosen to reflect functional tasks performed at an individual's natural pace, as previously validated in a similar protocol developed for elderly participants (André et al., 2016). This body‐weight–dependent task was selected because it reflects a functional movement commonly impaired in individuals with obesity (Cesanelli, Degens, et al., 2025), thereby providing high ecological validity. Although the external load is inherently higher in individuals with obesity, our intention was not to equalize load across groups but to examine performance during a real‐world functional task. During the test, the subjects were instructed to reach the maximal ankle ROM—marked on the wall—with both limbs and touching the heels to the ground at each repetition. The participants were barefoot, heels on the ground, knees extended, using their fingers touching a wall for balance. A reflective marker was placed on the malleolus, and a video was recorded at 88 frames per second using a high‐speed camera (acA1300‐75gc, Basler AG, Ahrensburg, Germany) in a sagittal position to track (Kinovea v.0.9.5, Charmant, J., & contributors) the vertical displacement of the ankle and to analyze the different phases of the calf raise exercise (i.e., isometric, concentric, and eccentric) (Cesanelli, Degens, et al., 2025). The concentric phase was identified as the upward movement of the ankle, the eccentric phase as the downward movement, and the isometric phase as the period when the ankle was stationary between movements.

### 2.5 Statistical analysis

Statistical analyses were performed using the IBM SPSS Statistics software package for Windows® (version 28.0.1.0). Data normality was assessed using the Shapiro–Wilk test. Group comparisons between CN and OB participants or animals were conducted using independent‐samples *t*‐tests. For isolated mouse muscles (EDL and SOL), comparisons included force production dynamics, active mechanical properties, and relaxation parameters derived from double exponential fits (k1, k2, and k1/k2 ratio). For human plantar flexors, MVC torque production and relaxation parameters were compared between groups. In the CRT, phase durations (eccentric, isometric, and concentric) were calculated and compared between groups. Effect size (ES) was determined based on Cohen's guidelines (Hopkins et al., 2009), with the standardized mean difference (d) for the pairwise comparisons interpreted as trivial (T), <0.20; small (S), 0.20–0.59; moderate (M), 0.60–1.19; large (L), 1.20–1.99; and very large (V), ≥2.00 (Fritz et al., 2012). Data are presented as mean ± standard deviation (SD). Statistical significance was set at *p* < 0.05.

## 3 RESULTS

### 3.1 Mouse study

OB mice were characterized by greater body mass than CN (29.2 ± 1.1 vs. 50.7 ± 2.6 g; *p* < 0.001, ES: 11.01—V), while there were no significant differences in SOL (OB: 1.18 ± 0.09 mm^2^; CN: 1.08 ± 0.18 mm^2^) and EDL (OB: 1.96 ± 0.05 mm^2^; CN: 1.89 ± 0.09 mm^2^) PCSA between the two groups (*p* > 0.05).

#### 3.1.1 Isolated muscles twitch contraction‐relaxation

There were no significant differences in peak absolute twitch force between groups in either SOL (OB: 32.3 ± 3.4 mN vs. CN: 33.2 ± 2.8 mN) or EDL (OB: 61.5 ± 7.4 mN vs. CN: 64.3 ± 6.7 mN) (*p* > 0.05 for both muscles), while the SOL RFD_E_ (OB: 690 ± 47 mN × s^−1^ vs. CN: 844 ± 90 mN × s^−1^) and RFD_L_ (OB: 346 ± 15 mN × s^−1^ vs. OB: 414 ± 29 mN × s^−1^) and EDL RFD_E_ (OB: 764 ± 56 mN × s^−1^ vs. CN: 1061 ± 37 mN × s^−1^) and RFD_L_ (OB: 422 ± 24 mN × s^−1^ vs. CN: 539 ± 37 mN × s^−1^) were significantly lower in OB than CN (*p* < 0.001, ES: 2.13).

**FIGURE 2:**
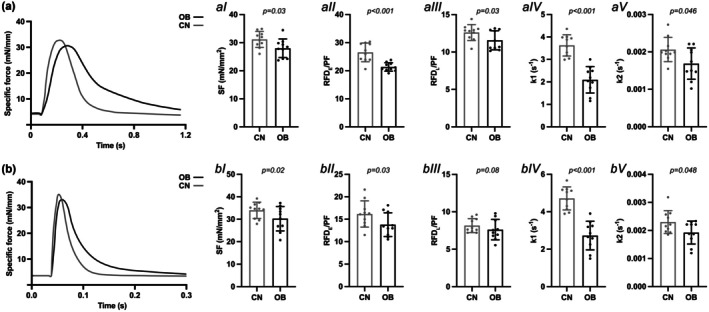
(a) Soleus (SOL) and (b) extensor digitorum longus (EDL) muscle twitch contraction force‐time curve example in obese (OB) and nonobese (CN) mice. Differences in (I) specific force (SF), rate of force development/peak force ratio (RFD/PF) for (II) early (_E_) and (III) late (_L_) phases, and force decay constants (IV) k1 and V) k2. Data are reported as mean ± SD. *p < 0.05; **p < 0.01; ***p < 0.001. Raw traces are shown for visual clarity; all analyses were performed on baseline‐subtracted data.

SOL of OB mice showed lower twitch SF (*p* = 0.03, ES: 1.01—M), RTD_E_/PF (*p* < 0.001, ES: 2.01—V), and RTD_L_/PF (*p* = 0.03, ES: 0.88—M) (Figure 2). The relaxation force decay constants k1 (*p* < 0.001, ES: 2.89—V) and k2 (*p* = 0.05, ES: 0.92—M) were lower in OB than CN (Figure 2). The k1/k2 ratio showed a non‐significant tendency toward lower values in OB compared to CN (*p* = 0.082, ES: 0.82; CN: 1622.83 ± 361.82 vs. OB: 1204.11 ± 621.27). HRT was significantly higher in OB than CN (*p* < 0.001, ES: 2.23; CN: 0.15 ± 0.01 s vs. OB: 0.17 ± 0.01 s). SOL of OB mice also showed a higher body mass × peak twitch force^−1^ ratio than CN (OB: 0.06 ± 0.01 g/mN; CN: 0.03 ± 0.00 g/mN; *p* < 0.001, ES: 4.24—V).

EDL of OB mice showed lower twitch SF (*p* = 0.02, ES: 0.99—M), RTD_E_/PF (*p* = 0.03, ES: 0.85—M) but not RTD_L_/PF (*p* = 0.08, ES: 0.59—S) (Figure 2). The relaxation force decay constant k1 (*p* < 0.001, ES: 2.86—V) and k2 (*p* = 0.05, ES: 0.84—M) were lower in OB than CN (Figure 2). The k1/k2 ratio was also lower in OB compared to CN (*p* = 0.009, ES: 1.31; CN: 2782.57 ± 1249.50 vs. OB: 1435.47 ± 745.39). HRT was significantly higher in OB than CN (*p* < 0.001, ES: 2.19; CN: 0.014 ± 0.002 s vs. OB: 0.020 ± 0.003 s). EDL of OB mice also showed a higher body mass × peak twitch^−1^ ratio than CN (OB: 0.04 ± 0.01 g/mN; CN: 0.02 ± 0.00 g/mN; *p* < 0.001, ES: 2.83—V).

#### 3.1.2 Isolated muscles tetanic contraction‐relaxation

On average, peak tetanic force was reached at 80 Hz for SOL and 100 Hz for EDL in both groups, and there were no significant differences between groups in either SOL (OB: 192 ± 30 mN vs. CN: 178 ± 22 mN) or EDL (OB: 280 ± 57 mN vs. CN: 303 ± 31 mN) (*p* > 0.05 for both muscles), while the SOL RFD_E_ (OB: 623 ± 110 mN × s^−1^ vs. CN: 771 ± 115 mN × s^−1^) and RFD_L_ (OB: 205 ± 28 mN × s^−1^ vs. CN: 265 ± 41 mN × s^−1^) and EDL RFD_E_ (OB: 2013 ± 477 mN × s^−1^ vs. CN: 2677 ± 416 mN × s^−1^) and RFD_L_ (OB: 757 ± 168 mN × s^−1^ vs. CN: 987 ± 100 mN × s^−1^) were significantly lower in OB than CN (*p* < 0.01, ES: 1.09).

**FIGURE 3:**
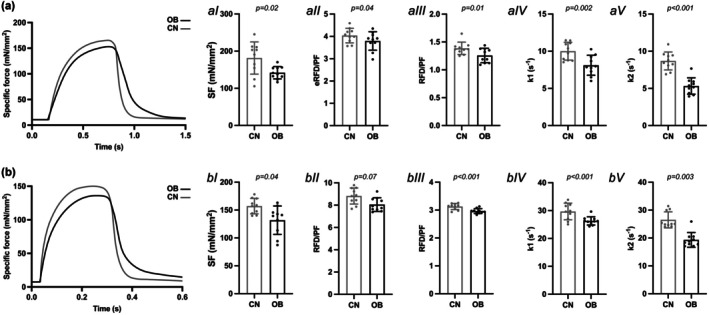
Soleus (SOL) and extensor digitorum longus (EDL) muscle peak tetanic contraction force‐time curve example in obese (OB) and nonobese (CN) mice. Differences in (I) specific force (SF), rate of force development/peak force ratio (RFD/PF) for (II) early (_E_) and (III) late (_L_) phases, and force decay constants IV) k1 and V) k2. Data are reported as mean ± SD. Raw traces are shown for visual clarity; all analyses were performed on baseline‐subtracted data.

SOL of OB mice showed lower tetanic SF (*p* = 0.02, ES: 0.96—M), RTD_E_/PF (*p* = 0.04, ES: 0.68—M), RTD_L_/PF (*p* < 0.01, ES: 1.12—M), and relaxation force decay constants, k_slow_ (*p* = 0.04, ES: 0.95; CN: 0.88 ± 0.08 vs. OB: 0.79 ± 0.11), k1 (*p* = 0.002, ES: 1.49—V) and k2 (*p* < 0.001, ES: 2.91– V) than CN (Figure 3). The k1/k2 ratio was also lower in OB compared to CN (*p* < 0.001, ES: 2.60; CN: 1.16 ± 0.11 vs. OB: 1.54 ± 0.17). HRT was significantly higher in OB than CN (*p* < 0.001, ES: 2.11; CN: 0.20 ± 0.02 s vs. OB: 0.26 ± 0.03 s). SOL of OB mice also showed a higher body mass × peak tetanic force^−1^ ratio than CN (OB: 0.31 ± 0.04 g/mN; CN: 0.16 ± 0.02 g/mN; *p* < 0.001, ES: 4.74—V).

EDL of OB mice showed lower tetanic SF (*p* = 0.04, ES: 0.77—M), RTD/PF (*p* = 0.07, ES: 1.35—L), RTD_L_/PF (*p* < 0.001, ES: 1.83—L), and relaxation force decay constants, k_slow_ (*p* = 0.07, ES: 1.33; CN: 2.86 ± 0.45 vs. OB: 2.23 ± 0.49), k1 (*p* < 0.001, ES: 1.76—L) and k2 (*p* = 0.003, ES: 1.54—L) than CN (Figure 3). The k1/k2 ratio was also lower in OB compared to CN (*p* = 0.040, ES: 0.878; CN: 1.15 ± 0.83 vs. OB: 1.33 ± 0.19). HRT was significantly higher in OB than CN (*p* = 0.002, ES: 1.63; CN: 0.043 ± 0.007 s vs. OB: 0.059 ± 0.012 s). EDL of OB mice showed also a higher body mass × peak tetanic force^−1^ ratio than CN (OB: 0.21 ± 0.05 g/mN; CN: 0.10 ± 0.05 g/mN; *p* < 0.001, ES: 3.05—V).

### 3.2 Human study

OB individuals were characterized by greater body mass (OB: 103.8 ± 7.6 kg; CN: 76.7 ± 6.9 kg; *p* < 0.001, ES: 3.49—V), body fat % (OB: 27.7 ± 2.8%; CN: 12.3 ± 2.5%; *p* < 0.001, ES: 5.79—V), and BMI than CN (OB: 31.9 ± 1.4 kg/m^2^; CN: 23.3 ± 1.5 kg/m^2^; *p* < 0.001, ES: 5.92—V).

#### 3.2.1 Maximal voluntary isometric contraction‐relaxation and calf raise phases

There were no significant differences in peak maximal voluntary isometric torque between OB and CN (OB: 183 ± 49 Nm; CN: 174 ± 33 Nm; *p* = 0.154, ES: 0.291—S).

**FIGURE 4:**
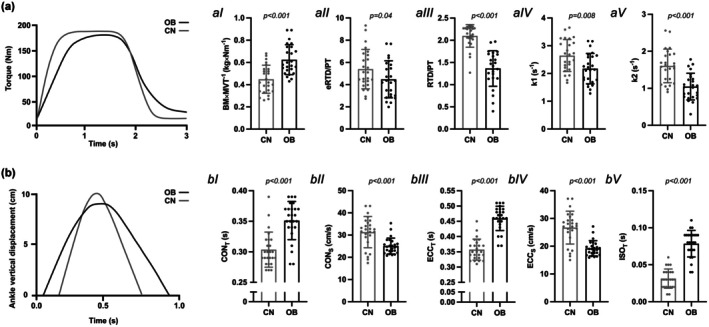
(a) Maximal voluntary isometric contraction (MVC) torque‐time curve example and (b) calf raise (CRT) ankle vertical displacement‐time curve example in individuals with obesity (OB) and normal weight (CN). Differences in (aI) body mass divided by maximal voluntary isometric torque (BM × MVT^−1^), rate of torque development/peak force ratio (RFD/PF) for (aII) early (E) and (aIII) late (L) phases, and force decay constants (aIV) k1 and (aV) k2. Differences in time spent in (bI) concentric (CON_T_), (bIII) eccentric (ECC_T_) and (bV) isometric (ISO_T_) phases and velocity of (bII) concentric (CON_S_) and (bIV) eccentric (ECC_S_) calf raise phases are reported. Data are reported as mean ± SD. Raw traces are shown for visual clarity; all analyses were performed on baseline‐subtracted data.

OB showed higher BM×MVT^−1^ (*p* < 0.001, ES: 1.35—L), lower RTD_E_/PT (*p* = 0.04, ES: 0.56—S), RTD_L_/PT (*p* < 0.001, ES: 1.79—L), and lower relaxation force decay constants, k1 (*p* = 0.008, ES: 0.71—M) and k2 (*p* < 0.001, ES: 1.11—L) than in CN (Figure 4). The k1/k2 ratio was also lower in OB compared to CN (*p* = 0.025, ES: 0.65; CN: 1.73 ± 0.62 vs. OB: 2.22 ± 0.86). HRT was significantly higher in OB than CN (*p* = 0.01, ES: 0.69; CN: 0.43 ± 0.07 s vs. OB: 0.49 ± 0.08 s). Each calf raise took more time in OB than CN, and particularly so in the eccentric (+28.4%) and concentric (+15.5%) phases (*p* < 0.001, ES: 1.60—L) (Figure 4).

## 4 DISCUSSION

The main observations of this study are that in both mice and men obesity was associated with slower muscle contractile properties and an elevated body‐mass‐to‐maximal‐force ratio. The latter reflects relative weakness that in mice was associated with a lower specific tension, suggesting that obesity is accompanied by a reduction in muscle quality. In men, the relative muscle weakness and slowing of the muscle contributed to a poorer performance of the calf raise test, where each calf raise, and particularly the concentric (raising) and eccentric (lowering), were slower in the individuals with obesity. Collectively, our results indicate that obesity is not only associated with relative muscle weakness but also slower contractile properties that contribute to impaired performance.

### 4.1 Impact of obesity on contractile properties

The results from our animal model of diet‐induced obesity indicate that obesity compromises the intrinsic force‐generating capacity of skeletal muscle. The lower specific force in the muscles of OB mice suggests a qualitative deterioration of the tissue that may be attributable to obesity‐driven (a) impairments in excitation‐contraction coupling (Tallis et al., 2018) and/or (b) structural remodeling, such as intramuscular lipid or fibrotic material accumulation (Cesanelli, Minderis, et al., 2025; Messa et al., 2020), that compromises the force and power generating capacity of the muscle tissue (Biltz et al., 2020; Cesanelli, Minderis, et al., 2025; Messa et al., 2020; Rahemi et al., 2015). Although PCSA did not differ significantly between groups, the lower specific force indicated contractile impairment.

Adiponectin signaling is blunted in OB muscle due to decreased AdipoR1 expression that in turn impairs downstream activation of calcium‐ and AMPK‐dependent pathways critical for mitochondrial and metabolic regulation (Tallis et al., 2018). At the cellular level, the reduced AMPK activity has been shown to reduce myocyte enhancer factor 2 (MEF2) signaling, and the reduction in calcineurin activity to lead to a suppression of calcium‐sensitive transcriptional pathways, both of which regulate genes involved in mitochondrial function and muscle plasticity (Tallis et al., 2018). However, these metabolic changes are unlikely to limit the force generation during the twitch and tetanic contractions assessed in our setup as they are brief and primarily ATP/PCr‐supported. Instead, the reduced specific force observed in OB muscle may be attributable to intrinsic deficits in excitation–contraction coupling, myofilament function and/or inter‐[9]and intra‐myocellular [32]lipid accumulation.

In support of problems with excitation‐contraction coupling, it has been reported that obesity is associated with impaired skeletal muscle calcium handling through multiple mechanisms. Notably, a reduced expression and activity of the sarcoplasmic/endoplasmic reticulum Ca^2+^‐ATPase (SERCA) will delay calcium reuptake into the sarcoplasmic reticulum. In addition, the lower ryanodine receptor (RyR1) expression and reductions in calcium‐buffering protein—such as calsequestrin—concentrations in the sarcoplasmic reticulum reduce calcium release in response to an action potential and diminish sarcoplasmic reticulum storage capacity, respectively. These changes collectively lower the amplitude and slow the kinetics of calcium transients, thereby impairing cross‐bridge cycling and contributing to reduced contractile force and slowing of muscle relaxation, particularly during high‐frequency tetanic stimulation where calcium cycling demands are elevated (Eshima, 2021; Tallis et al., 2018).

Beyond these changes in metabolism and calcium handling, structural and mechanical remodeling likely compounds the contractile deficits observed in OB muscles (Biltz et al., 2020; Cesanelli, Minderis, et al., 2025; Rahemi et al., 2015). For instance, obesity not only promotes intramuscular fat deposition, but also ECM remodeling and fibrosis that all can cause disruptions in sarcomeric alignment and mechanical force transmission (Biltz et al., 2020; Cesanelli, Minderis, et al., 2025; Rahemi et al., 2015). These ultrastructural changes blunt the efficiency of lateral force propagation and diminish the capacity to transmit fiber‐level force longitudinally to the tendon. Supporting this interpretation, previous work using the same OB model revealed marked histological alterations, including increased collagen deposition and lipid infiltration, consistent with changes in tissue mechanical properties (Cesanelli, Minderis, et al., 2025). Additionally, obesity‐related disruptions in sarcomeric alignment may broaden the effective force–length relationship, causing some fibers to operate off‐plateau and thereby reducing maximal force generation at a given muscle length (Tallis et al., 2018). These findings reinforce the notion that structural remodeling and concomitant metabolic alterations jointly impair overall muscle quality and force output capacity—and likely contribute to slower contractile properties in OB.

In sum, the lower specific force observed in OB muscle results from the convergence of metabolic–molecular dysfunction, impaired intracellular calcium handling, and structural–mechanical alterations, which compromise extracellular force transmission and overall muscle integrity. Although metabolic adaptations and fiber‐type changes in obesity can theoretically influence relaxation in both directions, the slowing observed in our study is empirically demonstrated under controlled in vitro isometric conditions. Several obesity‐related mechanisms can contribute to slowing of contractile properties, including impaired SERCA activity, reduced Ca^2+^ reuptake efficiency, lipid‐induced depression of cross‐bridge cycling, and increased ECM stiffness and viscosity (Rahemi et al., 2015; Tallis et al., 2018) [12]. These mechanisms provide physiologically plausible explanations for the slower relaxation kinetics observed in OB muscle.

We observed that obesity did not only have a negative impact on force production but also on the rate of relaxation. For the human plantar flexors and the mouse slow‐twitch SOL and fast‐twitch EDL, the double exponential decay function described the relaxation patterns during both the twitch and tetanus (and MVC) well (*R* ^2^: 0.94–0.99). It appeared that OB led particularly to a slowing of the fast initial decay (k1), which corresponds to the initial rapid decline in force following stimulation. This k1 phase is primarily determined by the rate of calcium reuptake into the sarcoplasmic reticulum, mediated by the SERCA pump (Sjåland et al., 2011) and the rate of cross‐bridge detachment, consistent with established descriptions of the fast relaxation component (Degens & Jones, 2020; Fitts, 2008; Westerblad & Allen, 1991). This slowing of the k1 phase of relaxation thus lends further support to an obesity‐induced slowing of calcium sequestration from the cytosol, delaying the uncoupling of actin–myosin cross‐bridges (Tallis et al., 2018). Moreover, energetic deficits resulting from reduced AMPK activity and reduced mitochondrial respiratory capacity can limit ATP availability for SERCA function and other ATP‐dependent relaxation processes, further exacerbating delays in the early relaxation phase (Sjåland et al., 2011; Tallis et al., 2018). While a slower rate of cross‐bridge detachment could also play a role, previous findings on fiber‐type shifts in obesity report no change in fiber type composition (Messa et al., 2020), or a modest slow‐to‐fast remodeling (Tallis et al., 2018) that would result, all else being the same, in faster rather than slower contractile kinetics.

The slower k2 phase reflects the slower, viscoelastic component of relaxation occurring once calcium concentrations in the cytosol have normalized (Cesanelli et al., 2024; Cesanelli, Minderis, et al., 2025; Mchugh et al., 1992; Smith & Barton, 2014). Such an increase in stiffness and viscosity may be the result of interstitial adipose accumulation, ECM thickening, and collagen cross‐linking seen in obesity (Biltz et al., 2020; Cesanelli, Minderis, et al., 2025; Rahemi et al., 2015). Another possibility worth exploring is alterations in titin, where for instance the lower passive tension in diaphragm fibers from patients with chronic obstructive pulmonary disease was associated with a longer elastic PEVK (proline‐glutamine‐valine‐lysine rich) segment of titin (Ottenheijm et al., 2006). The faster k2 in CN suggests that in healthy muscle, passive recoil is rapid and efficient, whereas in OB, the structurally altered matrix resists recoil, thereby prolonging relaxation.

Thus, while the k1 delay potentially reflects deficits in calcium‐dependent deactivation of cross‐bridges and ATP‐driven reuptake (Sjåland et al., 2011), the k2 prolongation points to the role of increased tissue viscosity and passive mechanical resistance due to obesity‐induced remodeling (Cesanelli, Minderis, et al., 2025).

It should also be considered that both k1 and k2 may be influenced by calcium‐dependent deactivation, cross‐bridge kinetics, and passive mechanical properties, such that obesity‐induced alterations affecting multiple components in parallel may manifest as a generalized slowing of relaxation across all analytical descriptors. Consistent with this interpretation, all relaxation metrics examined in the present study, including k_slow_, k1, k2, and HRT, converged in indicating slower relaxation kinetics in obesity, supporting the presence of a consistent physiological slowing.

In both SOL and EDL, twitch relaxation was conceivably driven almost entirely by the fast Ca^2+^‐dependent component (k_1_), consistent with classic descriptions of twitch decay being dominated by rapid SERCA‐mediated Ca^2+^ reuptake and cross‐bridge detachment (Bottinelli et al., 1996; Ruff, 1989; Stephenson & Williams, 1981). The slow (viscoelastic) component (k_2_) contributed minimally to twitch relaxation. This is also evident from the twitch traces, where an initial rapid decay is followed by a long, shallow tail that does not return fully to baseline within the brief twitch duration, matching the smaller k_2_ values (Figure 2 and Figure S1). In contrast, higher frequency tetani (e.g., 80‐150 Hz), k_1_ and k_2_ were of closer in magnitude, indicating that viscoelastic and series‐elastic elements (e.g., titin, connective tissue, and extracellular matrix structures) become substantially engaged mostly under sustained high‐force activation (Balnave & Allen, 1995; Lieber et al., 2017; Wang et al., 1993). Relaxation profiles at submaximal frequencies (20–50 Hz) further confirmed a progressive increase in k_2_ with activation duration, reflecting graded recruitment of the slow viscoelastic decay component (an example for SOL muscle is shown in Figure S1). Together, this pattern indicates that the intrinsic contractile machinery governs relaxation during brief twitches, whereas tissue composition and viscoelastic properties exert their greatest influence during sustained contractions, when slow relaxation pathways become progressively engaged. This pattern may also reflect the limited ability of double‐exponential fitting to dissociate overlapping physiological processes during short‐lived contractions, rather than the absence of passive or viscoelastic contributions to twitch relaxation.

To complement the exponential fitting approach, relaxation kinetics were also assessed using HRT, a model‐independent metric, across all contraction types. In addition, for tetanic contractions, relaxation was further characterized by quantifying the initial linear decay rate (K_slow_) from peak force to the onset of the rapid relaxation phase. The agreement between prolonged HRT and reduced k_slow_, k1, and k2 in OB muscle provides convergent evidence for consistently slowed relaxation kinetics, independent of curve‐fitting assumptions.

### 4.2 Impact of obesity on performance of the calf raise test

The above‐discussed findings reinforce the concept that obesity not only impairs active force production but also the rate of mechanical tension development and relaxation, through a combination of impaired excitation‐contraction coupling and an increase in viscosity and decrease in elasticity of the muscle tissue. This dual impairment could have significant implications for functional performance, particularly in cyclic tasks requiring rapid transitions between contraction and relaxation phases. Indeed, during the calf raise task, OB participants displayed both slower concentric (lifting) and eccentric (lowering) phases, despite instructions to move as fast as possible through full ROM. Although the calf raise is inherently load‐dependent, the direction and magnitude of the slowing parallel the deficits observed under load‐controlled conditions in both isolated mouse muscle and human isometric contractions. The delayed eccentric phase mirrors the prolonged k2 relaxation during the MVC relaxation. In addition, the higher BM × MVT^−1^ indicates that the obese muscles have to work at a slower part of the force velocity curve (Degens, 2019; Degens et al., 2021). In summary, the slower calf raises in the obese are thus a combination of slower contractile properties and the need to work at a slower part of the force‐velocity relationship.

The parallels in contractile dysfunction between mice and humans support a common mechanism by which obesity impairs muscle performance. Systemic inflammation, ectopic lipid accumulation, and impaired insulin signaling likely disrupt calcium homeostasis and myofibrillar function, slowing both contraction and relaxation. In parallel, obesity‐driven neuromuscular and musculotendinous remodeling delays force transmission and relaxation, compounding these effects (Cesanelli, Degens, et al., 2025; Tomlinson et al., 2016, 2021). Together, they have a significant negative impact on the ability to perform daily life activities, as reflected by the poorer performance in the calf raise test. Notably, such impairments were evident even in class I obesity without overt comorbidities, highlighting the early impact of excess adiposity on muscle quality and functional capacity. From a translational perspective, these results emphasize the need to address both strength and relaxation deficits to characterize the physiological response to obesity and to tailor proper rehabilitation and training programs for individuals with obesity.

### 4.3 Limitations

Despite the strengths of our integrative design, some limitations should be noted. In the human study, the maximal force generating capacity was assessed with voluntary contractions that at least in theory may be limited by motivational bias. In addition, in the human study, the absence of EMG recordings limited insights into neural versus muscular contributions to the observed impairments. In contrast, the animal model allowed precise ex vivo muscle function assessment but lacked in vivo functional dynamics. Still, the consistency between models supports the translational relevance of our findings.

While we did not perform fiber typing in these muscles, the slow phenotype of mouse SOL and the fast phenotype of mouse EDL are well established in the literature (James et al., 1995). One limitation is that both the murine and human cohorts consisted exclusively of males. This was done to reduce sex‐related biological variability (Koceva et al., 2024). Although in theory this may preclude inferences for females, the mechanisms and effects of obesity are similar in both sexes, with perhaps only a minor modification by sex‐specific hormone profiles. Nevertheless, it is of interest investigating whether obesity‐driven musculotendinous alterations differ between sexes.

Taken together, the combined use of linear, exponential, and time‐based relaxation metrics provides a conceptual framework to hypothesize how obesity‐induced alterations in calcium handling, cross‐bridge dynamics, and tissue mechanics interact to slow relaxation. Future studies should explore the cellular mechanisms underlying impaired relaxation, such as SERCA dysfunction, mitochondrial deficits, titin isoforms, and inflammation, and assess whether interventions or weight loss can restore the force generating capacity and the rates of contraction and relaxation. Incorporating functional tests that capture movement speed may also offer clinically relevant markers beyond traditional strength measures. Future work should also evaluate and compare alternative analytical models to more fully characterize relaxation kinetics and experimentally verify the relative contribution of calcium handling, cross‐bridge kinetics, and passive mechanical factors to the relaxation impairments identified here.

## 5 CONCLUSIONS

This study reveals that obesity not only impairs muscle force generating capacity but also results in slowing of the rate of force development and relaxation in both mice and men. These findings highlight the importance of considering not only force production but also contraction and relaxation kinetics as critical and underappreciated aspects of muscle dysfunction in obesity.

## FUNDING INFORMATION

No funding information provided.

## CONFLICT OF INTEREST STATEMENT

No conflicts of interest, financial or otherwise, are declared by the authors.

## ETHICS STATEMENT

All animal experiments were approved by the Lithuanian State Food and Veterinary Service (Ref. G2‐255).All human participants provided written informed consent after receiving detailed information about the study aims, procedures, and potential risks. The human study was approved by the Kaunas Regional Biomedical Research Ethics Committee (No. 2023‐BE10‐0001).

## Supporting information


Figure S1.

## Data Availability

The data that support the findings of this study are available upon request from the corresponding author.

## References

[phy270731-bib-0001] André, H.‐I. , Carnide, F. , Borja, E. , Ramalho, F. , Santos‐Rocha, R. , & Veloso, A. P. (2016). Calf‐raise senior: A new test for assessment of plantar flexor muscle strength in older adults: Protocol, validity, and reliability. Clinical Interventions in Aging, 11, 1661–1674. 10.2147/CIA.S115304 27895473 PMC5117878

[phy270731-bib-0002] Balasundaram, P. , & Daley, S. F. (2025). Public health considerations regarding obesity. In StatPearls. StatPearls Publishing. 34283488

[phy270731-bib-0003] Balnave, C. D. , & Allen, D. G. (1995). Intracellular calcium and force in single mouse muscle fibres following repeated contractions with stretch. The Journal of Physiology, 488(1), 25–36. 10.1113/jphysiol.1995.sp020943 8568662 PMC1156698

[phy270731-bib-0004] Biltz, N. K. , Collins, K. H. , Shen, K. C. , Schwartz, K. , Harris, C. A. , & Meyer, G. A. (2020). Infiltration of intramuscular adipose tissue impairs skeletal muscle contraction. The Journal of Physiology, 598, 2669–2683. 10.1113/JP279595 32358797 PMC8767374

[phy270731-bib-0005] Bottinelli, R. , Canepari, M. , Pellegrino, M. A. , & Reggiani, C. (1996). Force‐velocity properties of human skeletal muscle fibres: Myosin heavy chain isoform and temperature dependence. The Journal of Physiology, 495(2), 573–586. 10.1113/jphysiol.1996.sp021617 8887767 PMC1160815

[phy270731-bib-0006] Brooks, S. V. , & Faulkner, J. A. (1988). Contractile properties of skeletal muscles from young, adult and aged mice. The Journal of Physiology, 404, 71–82. 3253447 10.1113/jphysiol.1988.sp017279 PMC1190815

[phy270731-bib-0007] Cesanelli, L. , Degens, H. , Rifat Toper, C. , Kamandulis, S. , & Satkunskiene, D. (2025). Lower calf raise efficiency in obesity is partially related to higher triceps surae MTU passive stiffness, hysteresis, and reduced relative strength. Journal of Applied Physiology (1985), 138, 1066–1078. 10.1152/japplphysiol.00702.2024 40111915

[phy270731-bib-0008] Cesanelli, L. , Minderis, P. , Balnyte, I. , Ratkevicius, A. , Degens, H. , & Satkunskiene, D. (2025). Obesity‐driven musculotendinous remodeling impairs tissue resilience to mechanical damage. Cell and Tissue Research, 400, 287–302. 10.1007/s00441-025-03967-1 40163175

[phy270731-bib-0009] Cesanelli, L. , Minderis, P. , Degens, H. , & Satkunskiene, D. (2024). Passive mechanical properties of adipose tissue and skeletal muscle from C57BL/6J mice. Journal of the Mechanical Behavior of Biomedical Materials, 155, 106576. 10.1016/j.jmbbm.2024.106576 38744119

[phy270731-bib-0010] Corcos, D. M. , Chen, C.‐M. , Quinn, N. P. , Chen, C.‐. M. , McAuley, J. , & Rothwell, J. C. (1996). Strength in Parkinson's disease: Relationshp to rate of force generation and clinical status. Annals of Neurology, 39, 79–88. 10.1002/ana.410390112 8572671

[phy270731-bib-0011] Degens, H. (2019). Chapter 19—Human ageing: Impact on muscle force and power. In J. A. Zoladz (Ed.), Muscle and Exercise Physiology (pp. 423–432). Academic Press.

[phy270731-bib-0012] Degens, H. , Attias, J. , Evans, D. , Wilkins, F. , & Hodson‐Tole, E. (2021). The mobility limitation in healthy older people is due to weakness and not slower muscle contractile properties. PLoS One, 16, e0253531. 10.1371/journal.pone.0253531 34143856 PMC8213130

[phy270731-bib-0013] Degens, H. , & Jones, D. A. (2020). Are force enhancement after stretch and muscle fatigue due to effects of elevated inorganic phosphate and low calcium on cross bridge kinetics? Medicina (Kaunas, Lithuania), 56, 249. 10.3390/medicina56050249 32443826 PMC7279286

[phy270731-bib-0014] Dvornikov, A. V. , & Harris, S. P. (2025). Myosin‐binding protein C slows cardiac myofibril relaxation kinetics. The Journal of Physiology, 603, 5351–5368. 10.1113/JP289201 40936293 PMC13073943

[phy270731-bib-0015] Eshima, H. (2021). Influence of obesity and type 2 diabetes on calcium handling by skeletal muscle: Spotlight on the sarcoplasmic reticulum and mitochondria. Frontiers in Physiology, 12, 758316. 10.3389/fphys.2021.758316 34795598 PMC8592904

[phy270731-bib-0016] Fitts, R. H. (2008). The cross‐bridge cycle and skeletal muscle fatigue. Journal of Applied Physiology (1985), 104, 551–558. 10.1152/japplphysiol.01200.2007 18162480

[phy270731-bib-0017] Fokin, A. , Minderis, P. , Venckunas, T. , Lionikas, A. , Kvedaras, M. , & Ratkevicius, A. (2019). Myostatin dysfunction does not protect from fasting‐induced loss of muscle mass in mice. Journal of Musculoskeletal & Neuronal Interactions, 19, 342–353. 31475942 PMC6737554

[phy270731-bib-0018] Fritz, C. O. , Morris, P. E. , & Richler, J. J. (2012). Effect size estimates: Current use, calculations, and interpretation. Journal of Experimental Psychology. General, 141, 2–18. 10.1037/a0024338 21823805

[phy270731-bib-0019] Hausman, G. J. , Basu, U. , Du, M. , Fernyhough‐Culver, M. , & Dodson, M. V. (2014). Intermuscular and intramuscular adipose tissues: Bad vs. good adipose tissues. Adipocyte, 3, 242–255. 10.4161/adip.28546 26317048 PMC4550684

[phy270731-bib-0020] Hébert‐Losier, K. , Newsham‐West, R. J. , Schneiders, A. G. , & Sullivan, S. J. (2009). Raising the standards of the calf‐raise test: A systematic review. Journal of Science and Medicine in Sport, 12, 594–602. 10.1016/j.jsams.2008.12.628 19231286

[phy270731-bib-0021] Hopkins, W. G. , Marshall, S. W. , Batterham, A. M. , & Hanin, J. (2009). Progressive statistics for studies in sports medicine and exercise science. Medicine and Science in Sports and Exercise, 41, 3–13. 10.1249/MSS.0b013e31818cb278 19092709

[phy270731-bib-0022] Hunter, S. K. , Todd, G. , Butler, J. E. , Gandevia, S. C. , & Taylor, J. L. (2008). Recovery from supraspinal fatigue is slowed in old adults after fatiguing maximal isometric contractions. Journal of Applied Physiology, 105, 1199–1209. 10.1152/japplphysiol.01246.2007 18687979

[phy270731-bib-0023] James, R. S. , Altringham, J. D. , & Goldspink, D. F. (1995). The mechanical properties of fast and slow skeletal muscles of the mouse in relation to their locomotory function. The Journal of Experimental Biology, 198, 491–502. 10.1242/jeb.198.2.491 7699317

[phy270731-bib-0024] Koceva, A. , Herman, R. , Janez, A. , Rakusa, M. , & Jensterle, M. (2024). Sex‐ and gender‐related differences in obesity: From pathophysiological mechanisms to clinical implications. International Journal of Molecular Sciences, 25, 7342. 10.3390/ijms25137342 39000449 PMC11242171

[phy270731-bib-0025] Lieber, R. L. , Roberts, T. J. , Blemker, S. S. , Lee, S. S. M. , & Herzog, W. (2017). Skeletal muscle mechanics, energetics and plasticity. Journal of Neuroengineering and Rehabilitation, 14, 108. 10.1186/s12984-017-0318-y 29058612 PMC5651624

[phy270731-bib-0026] Maffiuletti, N. A. , Aagaard, P. , Blazevich, A. J. , Folland, J. , Tillin, N. , & Duchateau, J. (2016). Rate of force development: Physiological and methodological considerations. European Journal of Applied Physiology, 116, 1091–1116. 10.1007/s00421-016-3346-6 26941023 PMC4875063

[phy270731-bib-0027] Maffiuletti, N. A. , Jubeau, M. , Munzinger, U. , Bizzini, M. , Agosti, F. , De Col, A. , Lafortuna, C. L. , & Sartorio, A. (2007). Differences in quadriceps muscle strength and fatigue between lean and obese subjects. European Journal of Applied Physiology, 101, 51–59. 10.1007/s00421-007-0471-2 17476522

[phy270731-bib-0028] Mathern, R. M. , Anhorn, M. , & Uygur, M. (2019). A novel method to assess rate of force relaxation: Reliability and comparisons with rate of force development across various muscles. European Journal of Applied Physiology, 119, 291–300. 10.1007/s00421-018-4024-7 30367259

[phy270731-bib-0029] Mchugh, M. P. , Magnusson, S. P. , Gleim, G. W. , & Nicholas, J. A. (1992). Viscoelastic stress relaxation in human skeletal muscle. Medicine and Science in Sports and Exercise, 24, 1375. 1470021

[phy270731-bib-0030] Mehta, R. K. (2015). Impacts of obesity and stress on neuromuscular fatigue development and associated heart rate variability. International Journal of Obesity, 39, 208–213. 10.1038/ijo.2014.127 25042859

[phy270731-bib-0031] Messa, G. A. M. , Piasecki, M. , Hurst, J. , Hill, C. , Tallis, J. , & Degens, H. (2020). The impact of a high‐fat diet in mice is dependent on duration and age, and differs between muscles. The Journal of Experimental Biology, 223, jeb217117. 10.1242/jeb.217117 31988167 PMC7097303

[phy270731-bib-0032] Minderis, P. , Kilikevicius, A. , Baltusnikas, J. , Alhindi, Y. , Venckunas, T. , Bunger, L. , Lionikas, A. , & Ratkevicius, A. (2016). Myostatin dysfunction is associated with reduction in overload induced hypertrophy of soleus muscle in mice. Scandinavian Journal of Medicine & Science in Sports, 26, 894–901. 10.1111/sms.12532 26304113

[phy270731-bib-0033] Molenaar, J. P. , McNeil, C. J. , Bredius, M. S. , & Gandevia, S. C. (2013). Effects of aging and sex on voluntary activation and peak relaxation rate of human elbow flexors studied with motor cortical stimulation. Age, 35, 1327–1337. 10.1007/s11357-012-9435-5 22653296 PMC3705101

[phy270731-bib-0034] O'Brien, P. D. , Hinder, L. M. , Callaghan, B. C. , & Feldman, E. L. (2017). Neurological consequences of obesity. The Lancet Neurology, 16, 465–477. 10.1016/S1474-4422(17)30084-4 28504110 PMC5657398

[phy270731-bib-0035] Ottenheijm, C. A. C. , Heunks, L. M. A. , Hafmans, T. , van der Ven, P. , Benoist, C. , Zhou, H. , Labeit, S. , Granzier, H. L. , & Dekhuijzen, P. N. (2006). Titin and diaphragm dysfunction in chronic obstructive pulmonary disease. American Journal of Respiratory and Critical Care Medicine, 173, 527–534. 10.1164/rccm.200507-1056OC 16339921 PMC2662936

[phy270731-bib-0036] Rahemi, H. , Nigam, N. , & Wakeling, J. M. (2015). The effect of intramuscular fat on skeletal muscle mechanics: Implications for the elderly and obese. Journal of the Royal Society, Interface, 12, 20150365. 10.1098/rsif.2015.0365 26156300 PMC4535407

[phy270731-bib-0037] Robichaud, J. A. , Pfann, K. D. , Vaillancourt, D. E. , Comella, C. L. , & Corcos, D. M. (2005). Force control and disease severity in Parkinson's disease. Movement Disorders, 20, 441–450. 10.1002/mds.20350 15593316

[phy270731-bib-0038] Ruff, R. L. (1989). Calcium sensitivity of fast‐ and slow‐twitch human muscle fibers. Muscle & Nerve, 12, 32–37. 10.1002/mus.880120107 2473396

[phy270731-bib-0039] Sjåland, C. , Lunde, P. K. , Swift, F. , Munkvik, M. , Ericsson, M. , Lunde, M. , Boye, S. , Christensen, G. , Ellingsen, Ø. , Sejersted, O. M. , & Andersson, K. B. (2011). Slowed relaxation and preserved maximal force in soleus muscles of mice with targeted disruption of the Serca2 gene in skeletal muscle. The Journal of Physiology, 589, 6139–6155. 10.1113/jphysiol.2011.211987 21946846 PMC3286692

[phy270731-bib-0040] Smith, L. R. , & Barton, E. R. (2014). Collagen content does not alter the passive mechanical properties of fibrotic skeletal muscle in mdx mice. American Journal of Physiology Cell Physiology, 306, C889–C898. 10.1152/ajpcell.00383.2013 24598364 PMC4024713

[phy270731-bib-0041] Stephenson, D. G. , & Williams, D. A. (1981). Calcium‐activated force responses in fast‐ and slow‐twitch skinned muscle fibres of the rat at different temperatures. The Journal of Physiology, 317, 281–302. 10.1113/jphysiol.1981.sp013825 7310735 PMC1246789

[phy270731-bib-0042] Tallis, J. , James, R. S. , & Seebacher, F. (2018). The effects of obesity on skeletal muscle contractile function. Journal of Experimental Biology, 221, jeb163840. 10.1242/jeb.163840 29980597

[phy270731-bib-0043] Tomlinson, D. J. , Erskine, R. M. , Morse, C. I. , Pappachan, J. M. , Sanderson‐Gillard, E. , & Onambélé‐Pearson, G. L. (2021). The combined effects of obesity and ageing on skeletal muscle function and tendon properties in vivo in men. Endocrine, 72, 411–422. 10.1007/s12020-020-02601-0 33484409 PMC8128745

[phy270731-bib-0044] Tomlinson, D. J. , Erskine, R. M. , Morse, C. I. , Winwood, K. , & Onambélé‐Pearson, G. (2016). The impact of obesity on skeletal muscle strength and structure through adolescence to old age. Biogerontology, 17, 467–483. 10.1007/s10522-015-9626-4 26667010 PMC4889641

[phy270731-bib-0045] Wang, K. , McCarter, R. , Wright, J. , Beverly, J. , & Ramirez‐Mitchell, R. (1993). Viscoelasticity of the sarcomere matrix of skeletal muscles. The titin‐myosin composite filament is a dual‐stage molecular spring. Biophysical Journal, 64, 1161–1177. 10.1016/S0006-3495(93)81482-6 8494977 PMC1262434

[phy270731-bib-0046] Wearing, S. C. , Hennig, E. M. , Byrne, N. M. , Steele, J. R. , & Hills, A. P. (2006). The biomechanics of restricted movement in adult obesity. Obesity Reviews, 7, 13–24. 10.1111/j.1467-789X.2006.00215.x 16436099

[phy270731-bib-0047] Westerblad, H. , & Allen, D. G. (1991). Changes of myoplasmic calcium concentration during fatigue in single mouse muscle fibers. The Journal of General Physiology, 98, 615–635. 10.1085/jgp.98.3.615 1761971 PMC2229059

