# Supplementary figures and images for: From twitch to relaxation: Obesity dysregulates muscle contractile function
Source: Physiol Rep. 2026 Feb 15;14(4):e70731. doi: 10.14814/phy2.70731 (PMC12907575; doi:10.14814/phy2.70731)

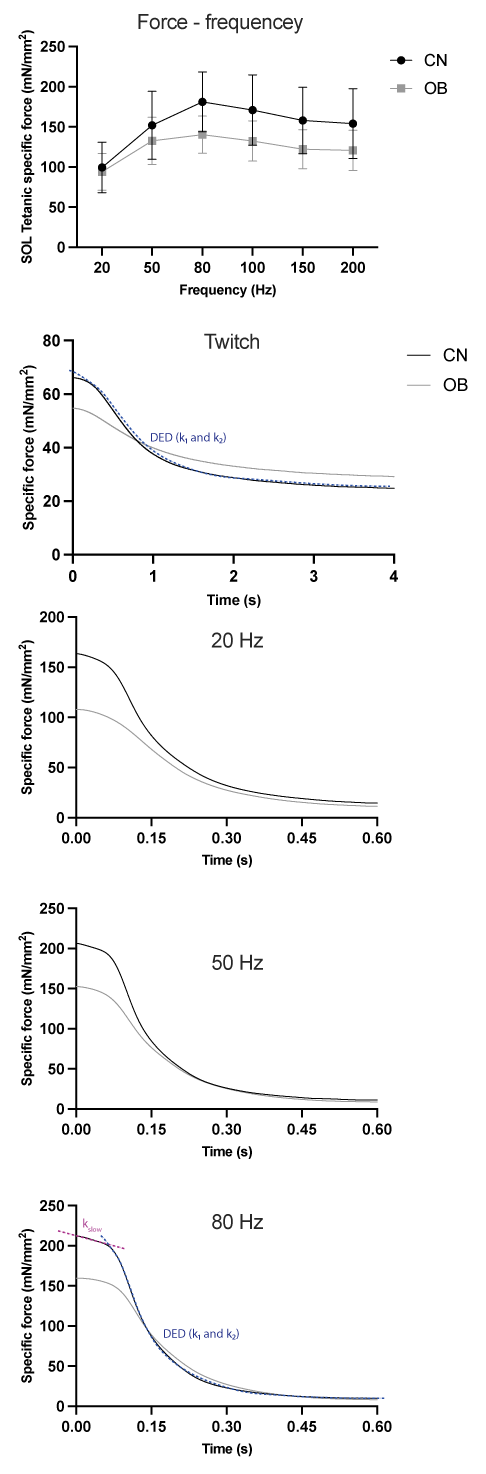

Supplement: Supplementary file 1 — Figure S1. [file PHY2-14-e70731-s001.tif]
